# Prediction of changes in suitable habitats for tea plants in China’s four major tea-producing regions based on machine learning models

**DOI:** 10.1371/journal.pone.0332382

**Published:** 2025-10-08

**Authors:** Wenqi Zhang, Lisha Yang, Yanxia Wang

**Affiliations:** College of Soil and Water Conservation, Southwest Forestry University, Kunming, Yunnan, China; National Cheng Kung University, TAIWAN

## Abstract

Under the background of ongoing global climate warming, clarifying the spatiotemporal dynamics of suitable habitats for tea plants and their potential impact on forest ecosystems is essential for promoting sustainable tea industry development and ecological conservation. This study integrated machine learning and geospatial analysis, using 14 climate, topographic, and soil variables to construct five models—Random Forest (RF), MaxEnt, Extreme Gradient Boosting (XGBoost), Support Vector Machine (SVM), and LightGBM. The best-performing RF model was selected to simulate current and future suitable habitats for tea plants across China’s Southwest Tea Region, Jiangnan Tea Region, Jiangbei Tea Region, and South China Tea Region under three Shared Socioeconomic Pathways (SSP1–2.6, SSP3–7.0, SSP5–8.5) at a 1 km spatial resolution. Based on this, the predicted habitat maps were overlaid with current forest distribution data to assess the potential risk of habitat expansion encroaching on forest land.Results show that:(1) Currently, the area of high and moderate suitable habitats in the four major tea-producing regions reaches 3.4401 million km^2^, accounting for 86.84% of the total tea cultivation area, and is mainly distributed in warm, humid regions with favorable ecological conditions;(2) Under future warming scenarios, suitable habitats are expected to shift northward overall, with significant increases in suitability in the Jiangnan Tea Region and Jiangbei Tea Region, edge expansion in the Southwest Tea Region, and stable patterns in the South China Tea Region;(3) Habitat expansion may pose regionally differentiated pressures on forest land, with significant increases in overlap with forest areas in the Southwest Tea Region and Jiangbei Tea Region under high-emission scenarios, indicating rising ecological conflict risks.This study provides scientific support and spatial insights for optimizing tea cultivation zoning, coordinating agricultural and forestry land use, and implementing climate-adaptive management strategies.

## 1. Introduction

Tea (*Camellia sinensis* (L.) Kuntze) is the most widely consumed beverage in the world after water, with China being the largest tea producer globally [1–3]. Tea plants are typically cultivated in monoculture plantations under humid climatic conditions, making them highly sensitive to climatic variations. Against the backdrop of prolonged drought and global warming in recent years, it is critical to explore whether the natural distribution of tea plants is shifting and whether human-driven expansion of tea plant cultivation could encroach upon forestlands, potentially leading to the loss of vital forest resources. Studying the current and future distribution of suitable habitats for tea plants, as well as their potential overlap with forest ecosystems, is essential for planning sustainable cultivation and balancing agroforestry development [4–6].

Previous studies have demonstrated that integrating machine learning with GIS-based spatiotemporal analysis has been widely applied in suitable habitat modeling, effectively overcoming the limitations of traditional statistical models in handling complex nonlinear relationships [7–10]. Among commonly used predictive algorithms are Random Forest (RF), Maximum Entropy (MaxEnt), Extreme Gradient Boosting (XGBoost), Support Vector Machine (SVM), and LightGBM. For example, Mi et al. (2017) used the RF model to predict potential distributions of cranes and other rare species across multiple regions [11]; Phillips et al. (2016) employed MaxEnt to assess suitable habitats for rangeland plants in Iran [12]; Du et al. (2025) applied the XGBoost–SHAP method to analyze plant community ecosystem services [13]; Dudík et al. (2006) used SVM to model ecological niches based on presence-only data [14]; and Buthelezi et al. (2024) used LightGBM to predict suitable forest vegetation zones [15]. While these models have shown excellent performance in predicting species distributions, they differ in their adaptability to small sample sizes, interpretation of climate responses, and extrapolation capability [16]. A comparative evaluation of model performance thus helps identify the most suitable model for projection under climate change scenarios.

As machine learning algorithms rely on data-driven paradigms, the accuracy and scale of prediction are primarily determined by input variables and sample quality. Existing research suggests that tea plant growth is influenced by a combination of complex factors. Climatic variables such as solar radiation, temperature, and precipitation are essential for assessing tea plant production potential on both global and regional scales [17]. Topographic features including elevation, slope, and aspect also play a significant role in determining the suitability and quality of tea plant cultivation [18–22]. Successful cultivation requires deep, well-drained acidic soils with pH values ranging from 4.5 to 5.5 [23–25]. These environmental factors—including soil, temperature, rainfall, sunlight, seasonality, and altitude—affect leaf morphology and the biosynthesis of key chemical compounds, ultimately shaping tea quality. Therefore, incorporating all relevant variables into importance ranking and selecting representative drivers is a feasible strategy to improve model performance.

Given China’s vast territory and diverse terrain, climate, and soil conditions, scientifically planning tea plant cultivation zones is vital for promoting mountain agriculture and forest-based economies, while maintaining ecosystem health. The expansion of suitable habitats for tea plants may also threaten biodiversity, as forests play a critical role in global carbon sequestration and contribute significantly to climate mitigation and sustainable development [26–28]. This study aims to estimate both current and future suitable habitats for tea plant cultivation and evaluate their potential encroachment on forest land. We compare the predictive performance of five mainstream machine learning models—RF, MaxEnt, XGBoost, SVM, and LightGBM—and use the best-performing model to simulate current and future suitable habitats. Spatial overlay analysis using GIS is then conducted to assess the risk of tea plant suitable habitat expansion encroaching upon forest ecosystems under different climate scenarios.

## 2. Research data

### 2.1. Study area and sample data

Tea plants thrive in warm and humid climates and are primarily distributed in southern China [29]. To better investigate the spatial distribution of suitable habitats for tea plants, this study focuses on China’s four major tea-producing regions, delineated according to the national tea zoning scheme [30,31]: (1) the Southwest Tea Region (Yunnan, Guizhou, Sichuan, and Chongqing), covering approximately 1.1452 million km^2^; (2) the Jiangnan Tea Region (Zhejiang, Anhui, Jiangsu, Hunan, Hubei, and Jiangxi), about 0.9177 million km^2^; (3) the Jiangbei Tea Region (including tea-producing areas in Longnan, southern Shaanxi, northern Sichuan, northern Hubei, northern Anhui, northern Jiangsu, southern Henan, and southeastern Shandong), roughly 0.1921 million km^2^; and (4) the South China Tea Region (Guangdong, Guangxi, Fujian, Taiwan, and Hainan), totaling about 0.6101 million km^2^ [32].

Presence records of tea plants were obtained from the Global Biodiversity Information Facility (GBIF, https://www.gbif.org), yielding 1,292 records of *Camellia sinensis*. After removing duplicate and erroneous records in ArcGIS 10.7, a 1 km × 1 km grid was applied nationwide. To reduce spatial autocorrelation, only one point per grid cell was retained. In total, 376 valid presence points were selected.

The distribution of these 376 tea plant presence points across the four major tea-producing regions is as follows: 132 in the Southwest Tea Region, 100 in the Jiangnan Tea Region, 119 in the South China Tea Region, and 13 in the Jiangbei Tea Region, with another 12 located outside the defined zones. The sample distribution across regions roughly corresponds to their respective areas—larger regions had more records, while smaller regions, such as the Jiangbei Tea Region, had fewer.

**Fig 1:**
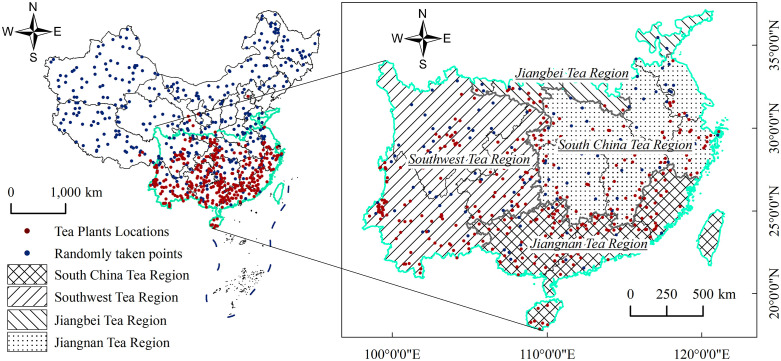
Location of the Four Major Tea-Producing Regions and Distribution of Tea Plant Sample Points in China.

To satisfy the requirement for both presence and absence data in machine learning models, the “disk” strategy proposed by Barbet-Massin et al. (2012) [33] was adopted. A total of 376 pseudo-absence points were randomly generated at least 10 km away from any presence point and then combined with the presence data, resulting in 752 sample points (Fig 1). Prior studies have also applied buffer distances of 10 km or greater in species distribution modeling [34,35], indicating the ecological and methodological relevance of this approach.

This map is based on the standard map provided by the Map Review Center of the Ministry of Natural Resources of China (Map Review Number: GS(2024)0650), with no modifications to the base map. Maps were produced by the authors.

### 2.2. Habitat factor data

Modern climatic data were obtained from the WorldClim database (https://www.worldclim.org/) and include 19 bioclimatic variables representing average conditions from 1970 to 2000. Future climate data were also sourced from WorldClim and derived from the BCC-CSM2-MR model developed by the National Climate Center (Beijing), which has demonstrated stable performance and strong simulation capability for Asian summer monsoon precipitation circulation. Three future climate scenarios were selected from two mid-term periods (2041–2060 [2050s] and 2081–2100 [2090s]), corresponding to SSP1–2.6, SSP3–7.0, and SSP5–8.5 of the Shared Socioeconomic Pathways (SSPs).

Digital elevation model (DEM) data for China were obtained from the 1 km resolution SRTM product available on the WorldClim website (https://worldclim.org). Slope and aspect layers were derived from the DEM data using ArcGIS. Landform data were sourced from the 1:4 million digital geomorphological dataset of China provided by the National Tibetan Plateau Data Center (TPDC, https://data.tpdc.ac.cn). Soil data were obtained from the high-resolution gridded national soil property dataset hosted by TPDC.

All environmental variables were resampled to a 1 km spatial resolution and standardized to the WGS_1984 geographic coordinate system and WGS_1984_UTM_Zone_49 projected coordinate system.

### 2.3. Land use data

The current forest cover data in China were obtained from the China Land Cover Dataset (CLCD), developed by the School of Remote Sensing at Wuhan University. The dataset spans 30 consecutive years (1990–2021) and is based on all available Landsat imagery processed via the Google Earth Engine (GEE) platform. It utilizes spatiotemporal features and a random forest classifier to generate land cover classifications, followed by a post-processing method incorporating spatiotemporal filtering and logical reasoning to improve the dataset’s temporal and spatial consistency. In this study, the 2021 land use data were selected to extract forest cover.

## 3. Methodology

**Fig 2:**
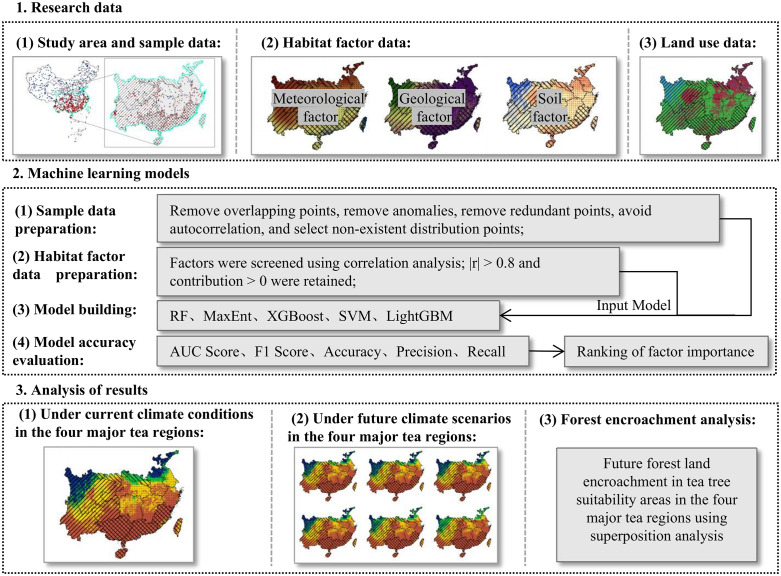
Technical Framework of the Study. Maps and diagrams were produced by the authors.

Based on the known distribution points of tea plants obtained from the Global Biodiversity Information Facility (GBIF), this study constructed five machine learning models using climatic, topographic, and soil habitat factors at a spatial resolution of 1 km as predictor variables. The model with the best performance was then selected to simulate and predict the habitat suitability of tea plants under historical average climate conditions, as well as under three future climate change scenarios: SSP1–2.6, SSP3–7.0, and SSP5–8.5. In addition, the study assessed the potential expansion of suitable tea planting areas and the associated risk of forest encroachment in the four major tea-producing regions of China. The technical framework of the study is shown in Fig 2.

### 3.1. Variable selection

**Fig 3:**
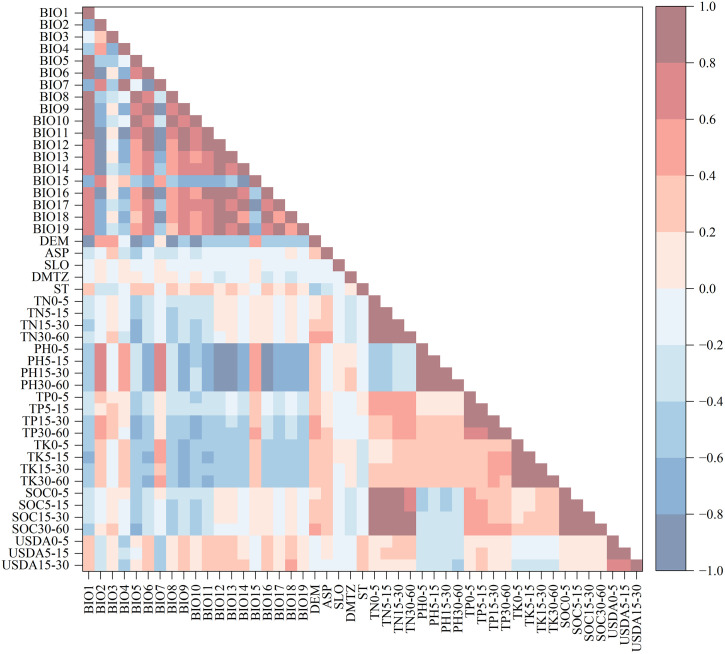
Pearson Correlation Heatmap Among Environmental Variables.

High collinearity among habitat factors may interfere with model prediction accuracy and the estimation of variable effects [36–38]. In this study, Pearson correlation analysis was conducted on all candidate environmental variables using the Python programming language, and the results are shown in Fig 3. Subsequently, all variables were initially input into the Random Forest (RF) model, and for each highly correlated variable pair with |r| > 0.8, the variable with a higher contribution to the model was retained based on importance evaluation. This process allowed for the removal of redundant variables and ensured that the variables input into the model were both representative and independent. The final 14 habitat factors retained are marked with a “✓” in Table 1, where the types and descriptions of all habitat variables are also detailed.

**Table 1:** Description of Habitat Factors Used in Modeling.

Factor Type	Code	Description	Unit	Retained
Meteorological Factor	BIO1	Annual Mean Temperature	°C	
	BIO2	Mean Diurnal Range	°C	
	BIO3	Isothermality	—	✓
	BIO4	Temperature Seasonality	°C	✓
	BIO5	Max Temperature of Warmest Month	°C	
	BIO6	Min Temperature of Coldest Month	°C	
	BIO7	Temperature Annual Range	°C	
	BIO8	Mean Temperature of Wettest Quarter	°C	
	BIO9	Mean Temperature of Driest Quarter	°C	
	BIO10	Mean Temperature of Warmest Quarter	°C	
	BIO11	Mean Temperature of Coldest Quarter	°C	
	BIO12	Annual Precipitation	mm	✓
	BIO13	Precipitation of Wettest Month	mm	
	BIO14	Precipitation of Driest Month	mm	
	BIO15	Precipitation Seasonality	—	✓
	BIO16	Precipitation of Wettest Quarter	mm	
	BIO17	Precipitation of Driest Quarter	mm	
	BIO18	Precipitation of Warmest Quarter	mm	
	BIO19	Precipitation of Coldest Quarter	mm	
Geological Factor	DEM	Digital Elevation Model	m	✓
	ASP	Slope direction	°	✓
	SLO	Elevation	°	✓
	DMTZ	Geomorphology	—	✓
Soil Factor	ST	Soil Thickness	cm	✓
	TN0–5	Nitrogen Content (0–5 cm)	g/kg	
	TN5–15	Nitrogen Content (5–15 cm)	g/kg	
	TN15–30	Nitrogen Content (15–30 cm)	g/kg	✓
	TN30–60	Nitrogen Content (30–60 cm)	g/kg	
	PH0-5	pH Value (0–5 cm)	—	
	PH5-15	pH Value (5–15 cm)	—	
	PH15-30	pH Value (15–30 cm)	—	
	PH30-60	pH Value (30–60 cm)	—	
	TP0–5	Phosphorus Content (0–5 cm)	g/kg	
	TP5–15	Phosphorus Content (5–15 cm)	g/kg	
	TP15–30	Phosphorus Content (15–30 cm)	g/kg	
	TP30–60	Phosphorus Content (30–60 cm)	g/kg	✓
	TK0–5	Potassium Content (0–5 cm)	g/kg	
	TK5–15	Potassium Content (5–15 cm)	g/kg	✓
	TK15–30	Potassium Content (15–30 cm)	g/kg	
	TK30–60	Potassium Content (30–60 cm)	g/kg	
	SOC0–5	Organic Carbon Content (0–5 cm)	g/kg	
	SOC5–15	Organic Carbon Content (5–15 cm)	g/kg	
	SOC15–30	Organic Carbon Content (15–30 cm)	g/kg	
	SOC30–60	Organic Carbon Content (30–60 cm	g/kg	
	USDA0–5	Soil Texture Type (0–5 cm)	—	
	USDA5–15	Soil Texture Type (5–15 cm)	—	✓
	USDA15–30	Soil Texture Type (15–30 cm)	—	✓

### 3.2. Machine learning model construction

This study employed five widely used machine learning algorithms to construct species distribution models, including Random Forest (RF), Maximum Entropy (MaxEnt), Support Vector Machine (SVM), Extreme Gradient Boosting (XGBoost), and LightGBM. These models are commonly applied in species distribution prediction and ecological suitability modeling due to their strong generalization abilities and robust handling of environmental variables, enabling them to effectively simulate complex nonlinear ecological responses.

Among them, both RF and XGBoost are ensemble learning methods with strong nonlinear modeling capabilities and noise resistance, making them suitable for ecological data with numerous variables and complex features. RF is insensitive to outliers and multicollinearity but has limited interpretability [39]; XGBoost incorporates regularization to enhance generalization but involves many parameters, making it prone to overfitting in small sample datasets [40]. MaxEnt is suited for presence-only data modeling, theoretically sound, and generates intuitive results, often used in species distribution modeling [41]. However, it is sensitive to variable collinearity and sampling bias, which may cause overestimation of suitable areas. SVM is ideal for high-dimensional and small-sample scenarios, offering clear classification boundaries and strong generalization ability [42], but it is computationally intensive and less scalable for large datasets due to its dependence on parameter tuning. LightGBM performs well in large-scale and high-dimensional data modeling, offering high training efficiency and prediction accuracy [43]. However, it is more sensitive to outliers and class imbalance, with weaker interpretability and some risk of overfitting.

All models were developed in Python 3.12 using popular open-source libraries such as Scikit-learn, XGBoost, and LightGBM. ArcGIS was used to extract species presence points, background points, and environmental variables. Of all presence points, 70% were randomly selected as the training set, and the remaining 30% were used for model evaluation. Hyperparameters were optimized using GridSearchCV with five-fold cross-validation, and the final settings were as follows:

- RF: n_estimators = 70, max_depth = 10, max_features = ‘sqrt’, min_samples_split = 2, min_samples_leaf = 4;
- MaxEnt: C = 0.1, solver = ‘lbfgs’;
- SVM: C = 10, kernel = ‘linear’, gamma = ‘scale’;
- XGBoost: learning_rate = 0.1, max_depth = 10, n_estimators = 80, subsample = 1.0, colsample_bytree = 0.8;
- LightGBM: learning_rate = 0.05, max_depth = 10, n_estimators = 80, num_leaves = 31.

### 3.3. Model accuracy evaluation

To comprehensively evaluate the performance of machine learning models in predicting the suitability of tea plants, this study adopted multiple evaluation metrics: AUC Score, F1 Score, Accuracy, Precision, and Recall. Each metric captures different aspects of model performance, allowing for a thorough understanding of the strengths and weaknesses of each approach.

1. **AUC Score**: The Area Under the Curve (AUC) is a key indicator that measures a model’s ability to distinguish between classes across all possible thresholds, with values ranging from 0.5 to 1.0 [44–46]. Specifically, an AUC of 0.5–0.6 indicates weak prediction ability; 0.6–0.7 suggests suboptimal performance; 0.7–0.8 is considered moderate; 0.8–0.9 is good; and 0.9–1.0 reflects excellent predictive capability [47,48].
2. **F1 Score**: The F1 score is the harmonic mean of Precision and Recall, as shown in Equation (1):

F1=2 × (Precision × Recall) / (Precision + Recall)  (1)

It provides a balanced view of overall model performance and is particularly suitable for evaluating imbalanced datasets, where relying solely on accuracy could be misleading.

1. **Accuracy**: Accuracy measures the proportion of correctly predicted samples among all samples, as shown in Equation (2):

Accuracy = (TP + TN) / (TP + TN + FP + FN) (2)

where TP is True Positive, TN is True Negative, FP is False Positive, and FN is False Negative.

1. **Precision**: Precision is the proportion of true positive predictions among all positive predictions, as shown in Equation (3):

Precision = TP / (TP + FP) (3)

It reflects the model’s ability to avoid false alarms.

1. **Recall**: Recall is the proportion of true positive samples correctly identified by the model, as shown in Equation (4):

Recall = TP / (TP + FN) (4)

This metric indicates the model’s sensitivity to the target class.

By integrating these evaluation metrics, this study aims to comprehensively assess model performance in tea plant suitability prediction and provide a theoretical foundation and practical reference for future model selection and optimization.

### 3.4. Suitable Habitat Map Generation and Classification

To generate the suitability probability map for tea plants, a point conversion was first applied to the 1 km resolution raster map of China. The trained machine learning models were then used to calculate the suitability probability for each point. These results were imported into ArcGIS, where a point-to-raster conversion was conducted to produce a continuous spatial probability map. This approach has been widely applied in species distribution modeling and suitability prediction, effectively capturing the spatial distribution patterns of potential habitats [49,50]. Based on this, the classification method proposed by Tang Junxian et al. [51] was adopted to categorize tea plant suitability levels. Using the Natural Breaks (Jenks) classification method, the suitability probabilities were divided into four levels: high habitability area, habitability area, middle habitability area, and low habitability area.

### 3.5. Overlay analysis

To visually assess the potential land-use conflicts between future climate-driven expansion of tea plant habitability areas and existing forest ecosystems, this study employed ArcGIS to conduct spatial overlay analysis. The modeled tea plant habitability maps under current and future climate scenarios were overlaid with forest land cover layers derived from China’s land use data. This approach has been widely applied in studies on species distribution shifts and land-use conflicts, and has proven effective in identifying potential threats to ecologically sensitive areas [52–54].

Specifically, the areas classified as high habitability areas and habitability areas were merged and defined as the primary habitability area for tea plant cultivation. These zones were spatially intersected with forest land layers to extract the overlapping areas under each climate scenario. Using the current overlap area as a reference, the future overlap area and its percentage relative to the current forest area were calculated, thereby quantifying the potential expansion pressure of tea plant cultivation suitability on forest ecosystems.

## 4. Results

### 4.1. Accuracy and factor importance evaluation of machine learning models

**Fig 4:**
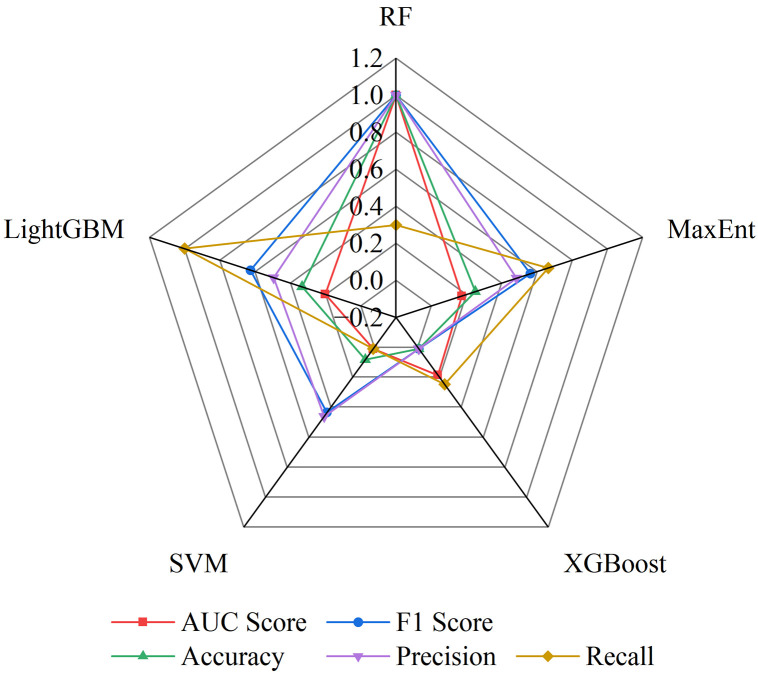
Radar Chart of Overall Performance of Different Models Across Five Evaluation Metrics.

This study employed five machine learning models—Random Forest (RF), Maximum Entropy (MaxEnt), Extreme Gradient Boosting (XGBoost), Support Vector Machine (SVM), and Light Gradient Boosting Machine (LightGBM)—to predict the habitat suitability of tea plants. The performance of each model was evaluated using the following metrics: Area Under the Curve (AUC), F1 Score, Accuracy, Precision, and Recall. The evaluation results for each model across the five metrics are presented in Table 2, and the overall performance comparison is illustrated in the radar chart shown in Fig 4.

**Table 2:** Performance Metrics of Machine Learning Models.

Model	AUC	F1-score	Accuracy	Precision	Recall
**RF**	0.9431	0.9143	0.9071	0.8819	0.9492
**MaxEnt**	0.9076	0.8846	0.8673	0.8214	0.9583
**XGBoost**	0.9081	0.8465	0.8540	0.7647	0.9479
**SVM**	0.9001	0.8760	0.8584	0.8188	0.9417
**LightGBM**	0.9088	0.8889	0.8717	0.8227	0.9667

**Fig 5:**
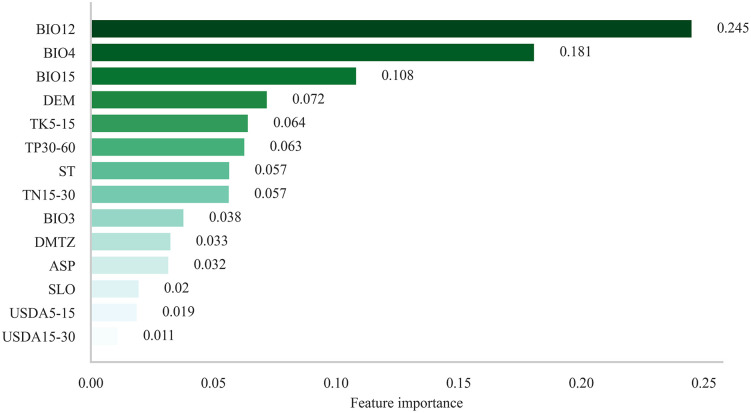
Variable Importance Ranking of the Random Forest Model. The meanings of the environmental factor codes are shown in Table 1.

As shown in Table 2 and Fig 4, all five machine learning models performed well; however, the Random Forest model clearly outperformed the others in terms of stability and predictive performance. Therefore, the subsequent analyses and predictions adopt the Random Forest model as the primary predictive tool.In addition, the variable importance analysis of the Random Forest model indicated that all 14 environmental variables had importance scores exceeding 1%, with the top eight variables collectively contributing 84.7%, as illustrated in Fig 5. These results suggest that these variables are the main driving factors determining the distribution of suitable habitats for tea plants in China.

### 4.2. Distribution of tea plant suitable areas in the four major tea-producing regions of China under current climate conditions

**Fig 6:**
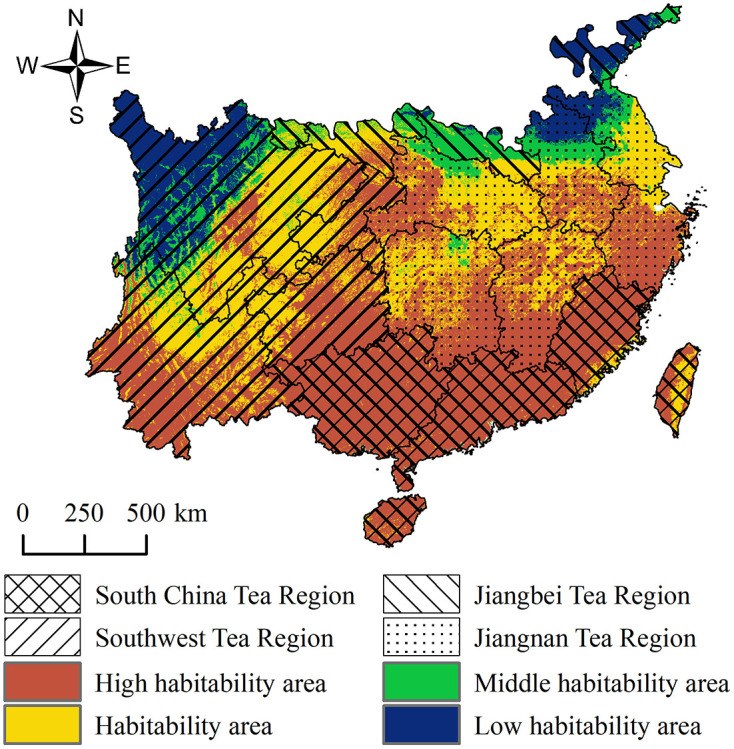
Distribution of Tea Plant Suitable Areas in the Four Major Tea Regions of China under Current Climate Conditions. Maps were produced by the authors.

Under current climate conditions, tea plant habitability areas are widely distributed across the four major tea-producing regions of China, primarily concentrated in high habitability areas and habitability areas (Fig 6). Model predictions indicate that the total area of these two categories within the four regions is 2.3065 million km^2^, accounting for approximately 80.51% of the total area, suggesting that the present climate is generally favorable for tea plant growth.

Among the four regions, high habitability areas cover 1.4542 million km^2^ (50.76%), representing the dominant habitability level for tea plants. These areas are mainly distributed in the Southwest Tea Region, South China Tea Region, and parts of the Jiangnan Tea Region. Habitability areas extend over 0.8523 million km^2^ (29.75%), generally forming buffer zones surrounding high habitability zones. Middle habitability areas occupy 0.2527 million km^2^ (8.82%), mostly located north of the Yangtze River and along the northwestern margins, while low habitability areas total 0.3058 million km^2^ (10.67%), mainly found in colder or drier regions such as plateaus and northern areas.

From a regional perspective:

- The Southwest Tea Region is the core zone for high suitability, with 0.4307 million km^2^ of high habitability areas and 0.4198 million km^2^ of habitability areas, together comprising 74.26% of the region’s total area. The complex terrain and the transition from subtropical monsoon to plateau monsoon climate create favorable conditions for diverse tea plant varieties.
- The Jiangnan Tea Region has a mild and humid climate, with 0.4367 million km^2^ of high habitability areas (47.58%) and 0.3452 million km^2^ of habitability areas (37.62%), totaling 85.20%. As a traditional tea-producing region, it is densely cultivated and holds further potential for improving tea plantation quality.
- The Jiangbei Tea Region exhibits relatively low overall habitability due to its higher latitude and colder climate, with 0.124 million km^2^ (6.46%) of high habitability areas and 0.576 million km^2^ (29.98%) of habitability areas. Middle habitability areas reach 0.733 million km^2^, accounting for more than one-third of the region, suggesting a certain degree of development potential.
- The South China Tea Region enjoys the most favorable climate, with 0.5745 million km^2^ of high habitability areas (94.16%) and 0.297 million km^2^ of habitability areas (4.87%), indicating that nearly the entire region falls into the high habitability category, making it the core production area of China’s tea industry.

Overall, the distribution of tea plant habitability areas across the four regions exhibits a southeast–northwest gradient, with high habitability areas concentrated in warm, humid, low-latitude zones, while low habitability areas are mainly located in marginal climates less suitable for tea plant cultivation.

### 4.3 Distribution and changes of tea plant suitable areas in the four major tea regions of China under future climate scenarios

**Fig 7:**
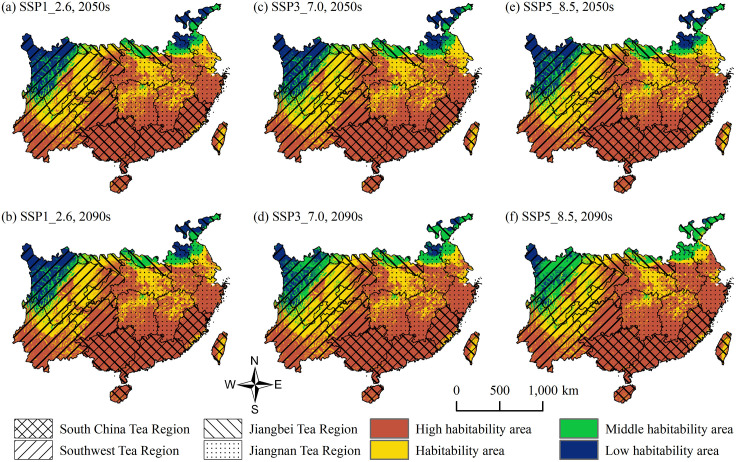
Changes in Tea Plant Suitable Areas across China’s Four Major Tea Regions under Future Climate Scenarios. **(a)** SSP1_2.6, 2050s; **(b)** SSP1_2.6, 2090s; **(c)** SSP3_7.0, 2050s; **(d)** SSP3_7.0, 2090s; **(e)** SSP5_8.5, 2050s; **(f)** SSP5_8.5, 2090s. Maps were produced by the authors.

Under the two future periods and three climate scenarios, the potential suitable habitats for tea plants in China’s four major tea-producing regions are shown in Fig 7, with detailed changes in area and proportion presented in Table 3.

**Table 3:** Changes in Area and Proportion of Suitable Habitats for Tea Plants in China’s Four Major Tea-Producing Regions Under Future Climate Scenarios.

Tea Region	Climate scenario	High habitability area(×10^4^ km^2^)	High habitability area (%)	Habitability area(×10^4^ km^2^)	Habitability area (%)	Middle habitability area(×10^4^ km^2^)	Middle habitability area (%)	Low habitability area(×10^4^ km^2^)	Low habitability area (%)
Southwest Tea Region	Current	43.07	37.60%	41.98	36.66%	9.24	8.06%	20.24	17.67%
	SSP1_2.6,2050s	46.02	40.18%	39.74	34.70%	9.76	8.52%	19.00	16.59%
	SSP1_2.6,2090s	48.20	42.09%	37.78	32.99%	9.98	8.71%	18.56	16.21%
	SSP3_7.0,2050s	41.67	36.39%	42.39	37.01%	11.10	9.70%	19.36	16.90%
	SSP3_7.0,2090s	43.99	38.41%	41.38	36.14%	16.56	14.46%	12.59	11.00%
	SSP5_8.5,2050s	44.36	38.74%	40.64	35.49%	10.77	9.40%	18.75	16.37%
	SSP5_8.5,2090s	45.54	39.76%	40.33	35.21%	20.44	17.85%	8.22	7.18%
Jiangnan Tea Region	Current	43.67	47.58%	34.52	37.62%	8.12	8.84%	5.47	5.96%
	SSP1_2.6,2050s	54.87	59.80%	28.47	31.03%	5.23	5.70%	3.19	3.48%
	SSP1_2.6,2090s	56.65	61.73%	26.59	28.98%	5.60	6.10%	2.93	3.19%
	SSP3_7.0,2050s	52.72	57.46%	29.29	31.92%	6.04	6.58%	3.72	4.05%
	SSP3_7.0,2090s	58.16	63.38%	25.62	27.92%	5.93	6.46%	2.05	2.24%
	SSP5_8.5,2050s	53.82	58.64%	28.70	31.28%	5.89	6.42%	3.35	3.65%
	SSP5_8.5,2090s	64.43	70.21%	22.03	24.01%	5.17	5.63%	0.13	0.15%
Jiangbei Tea Region	Current	1.24	6.46%	5.76	29.98%	7.33	38.18%	4.87	25.38%
	SSP1_2.6,2050s	3.30	17.18%	5.74	29.88%	6.50	33.85%	3.67	19.09%
	SSP1_2.6,2090s	2.85	14.86%	5.76	29.97%	6.99	36.41%	3.60	18.77%
	SSP3_7.0,2050s	2.69	13.99%	5.74	29.91%	6.90	35.95%	3.87	20.15%
	SSP3_7.0,2090s	3.51	18.29%	6.07	31.59%	8.05	41.92%	1.57	8.20%
	SSP5_8.5,2050s	2.75	14.34%	5.73	29.86%	7.05	36.73%	3.66	19.07%
	SSP5_8.5,2090s	4.44	23.13%	6.53	33.98%	7.71	40.12%	0.53	2.77%
South China Tea Region	Current	57.45	94.16%	2.97	4.87%	0.59	0.96%	0.00	0.00%
	SSP1_2.6,2050s	58.08	95.20%	2.93	4.80%	0.00	0.00%	0.00	0.00%
	SSP1_2.6,2090s	58.09	95.20%	2.93	4.80%	0.00	0.00%	0.00	0.00%
	SSP3_7.0,2050s	57.91	94.91%	3.10	5.08%	0.00	0.00%	0.00	0.00%
	SSP3_7.0,2090s	57.99	95.04%	3.03	4.96%	0.00	0.00%	0.00	0.00%
	SSP5_8.5,2050s	57.99	95.05%	3.02	4.95%	0.00	0.00%	0.00	0.00%
	SSP5_8.5,2090s	58.06	95.15%	2.96	4.84%	0.00	0.00%	0.00	0.00%

In the Southwest Tea Region, the area of high habitability areas shows an overall increasing trend across the six climate scenarios. In the SSP1–2.6 2090s scenario, the proportion reaches 42.09%, representing an increase of 4.49% compared with the current climate (see Section 4.2). The proportion of habitability areas slightly decreases, fluctuating between 32.99% and 37.01%. In the SSP5–8.5 2090s scenario, the proportion of middle habitability areas rises to 17.85%, the highest among all scenarios, indicating that under high-temperature and high-emission conditions, ecological suitability in marginal zones may decline. Given the diverse topography and ecological vulnerability of this region, it is recommended to strengthen forest conservation and sustainable tea plantation management, promote tea–forest symbiosis models and modern agricultural practices, so as to balance ecological and economic benefits.

In the Jiangnan Tea Region, rising temperatures and increased precipitation in future climate scenarios will lead to a significant expansion of high habitability areas. In the SSP5–8.5 2090s scenario, the proportion reaches 70.21%, an increase of 22.63% compared with the current climate; meanwhile, the proportion of habitability areas decreases to 24.01%, and middle and low habitability areas decline markedly. This suggests that suitability will become highly concentrated in high habitability areas. However, the risks of extreme climate events such as heavy rainfall and flooding should be considered. Strengthening soil and water conservation, shelterbelt construction, and drainage systems is advised to enhance the climate resilience of tea plantations.

In the Jiangbei Tea Region, overall suitability improves significantly with climate warming. In the SSP5–8.5 2090s scenario, the proportion of high habitability areas reaches 23.13%, an increase of 16.67% compared with the current climate, while low habitability areas decrease by 22.61%. Middle habitability areas also increase under multiple scenarios, indicating a clear improvement in overall suitability. Introducing cold- and drought-resistant cultivars, optimizing irrigation systems, and enhancing soil water retention are recommended to improve the adaptability and stability of tea plantations.

In the South China Tea Region, the proportion of high habitability areas changes very little across the six scenarios, peaking at 95.20%. The proportion of habitability areas slightly declines to 4.84%–5.08%, and middle and low habitability areas nearly disappear, indicating a highly stable overall suitability. It is suggested to focus on disaster prevention and ecological optimization, improving drainage, flood control, and windbreak facilities to enhance the disaster resilience of tea plantations.

Overall, under different future climate scenarios, the suitability patterns of tea plants in China’s four major tea-producing regions are projected to exhibit a general trend of “stability in the south, improvement in the north, and expansion in the east.” Suitability in the Jiangnan and Jiangbei Tea Regions is expected to improve significantly, the Southwest Tea Region will maintain its advantages, and the South China Tea Region will remain at a consistently high level of suitability. These trends provide important references for adjusting the spatial layout of tea cultivation, optimizing planting structures, and improving the industry’s climate adaptation strategies.

### 4.4. Potential forest encroachment by major suitable areas for tea plants in China’s four major tea regions under future climate scenarios

**Fig 8:**
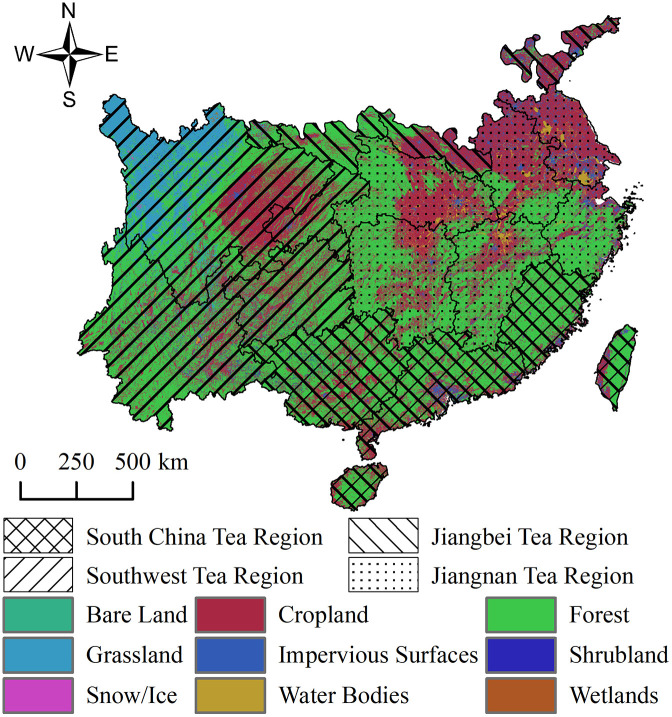
Land-Use Types in the Four Major Tea Regions of China. Land-use data were obtained from the China Land Cover Dataset (CLCD, 1990–2021) developed by the School of Remote Sensing, Wuhan University, based on Landsat imagery processed via Google Earth Engine. Maps were produced by the authors.

**Fig 9:**
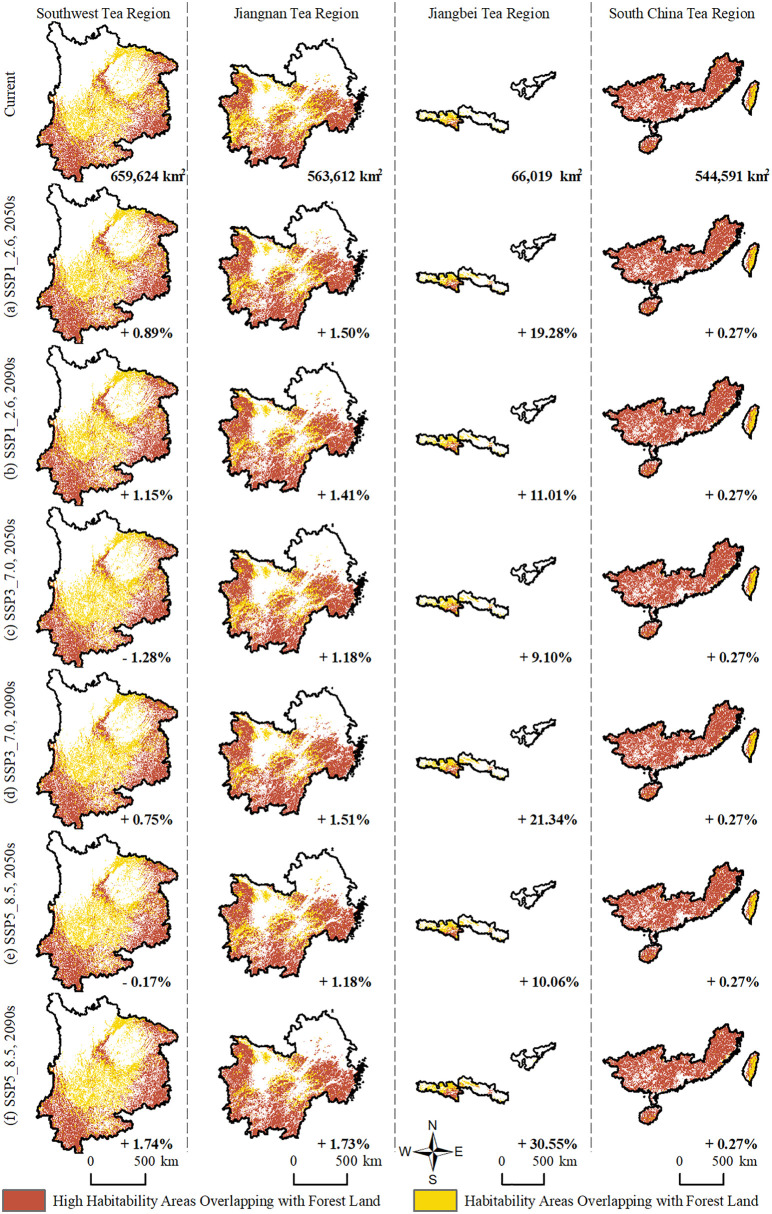
Overlap between Primary Habitability Areas for Tea Plants and Forest Land in China’s Four Major Tea Regions. Forest land data were obtained from the China Land Cover Dataset (CLCD, 1990–2021) developed by the School of Remote Sensing, Wuhan University. Maps were produced by the authors.

The spatial expansion of tea plant habitability areas is not only driven by climate change but may also pose potential threats to existing ecosystem structures. In China’s four major tea-producing regions, forests constitute the dominant land use type—particularly in the Southwest Tea Region, South China Tea Region, and Jiangnan Tea Region—where they show a high degree of spatial overlap with high habitability areas (Fig 8). Under future climate scenarios, the changes in the area and proportion of forest land encroached by primary habitability areas for tea plants are presented in Table 4, while the spatial overlap distribution is illustrated in Fig 9.

**Table 4:** Changes in Area and Proportion of Forest Land Occupied by Primary Habitability Areas for Tea Plants in China’s Four Major Tea-Producing Regions Under Future Climate Scenarios.

Tea Region	Climate scenario	Overlap Area between High Habitability Areas and Forest Land (×10^4^ km^2^)	Overlap Area between Habitability Areas and Forest Land(×10^4^ km^2^)	Overlap Area between Primary Habitability Areas and Forest Land(×10^4^ km^2^)	Change in Area Compared to Current Climate Conditions(×10^4^ km^2^)	Percentage Change in Area Compared to Current Climate Conditions (%)
Southwest Tea Region	Current	27.86	22.54	50.41	——	——
	SSP1_2.6,2050s	29.62	21.23	50.86	0.45	0.89%
	SSP1_2.6,2090s	30.80	20.19	50.99	0.58	1.15%
	SSP3_7.0,2050s	27.21	22.55	49.77	−0.64	−1.28%
	SSP3_7.0,2090s	28.72	22.07	50.79	0.38	0.75%
	SSP5_8.5,2050s	28.68	21.64	50.32	−0.09	−0.17%
	SSP5_8.5,2090s	29.77	21.52	51.28	0.88	1.74%
Jiangnan Tea Region	Current	28.21	14.87	43.07	——	——
	SSP1_2.6,2050s	32.68	11.04	43.72	0.65	1.50%
	SSP1_2.6,2090s	33.60	10.07	43.68	0.61	1.41%
	SSP3_7.0,2050s	32.28	11.30	43.58	0.51	1.18%
	SSP3_7.0,2090s	34.12	9.60	43.72	0.65	1.51%
	SSP5_8.5,2050s	32.59	10.99	43.58	0.51	1.18%
	SSP5_8.5,2090s	36.27	7.55	43.82	0.75	1.73%
Jiangbei Tea Region	Current	1.07	3.98	5.05	——	——
	SSP1_2.6,2050s	1.77	4.25	6.02	0.97	19.28%
	SSP1_2.6,2090s	1.54	4.17	5.71	0.66	11.01%
	SSP3_7.0,2050s	1.45	4.12	5.56	0.52	9.10%
	SSP3_7.0,2090s	1.90	4.33	6.23	1.19	21.34%
	SSP5_8.5,2050s	1.46	4.21	5.67	0.63	10.06%
	SSP5_8.5,2090s	2.37	4.41	6.78	1.73	30.55%
South China Tea Region	Current	39.40	2.22	41.62	——	——
	SSP1_2.6,2050s	39.53	2.19	41.73	0.11	0.27%
	SSP1_2.6,2090s	39.55	2.18	41.73	0.11	0.27%
	SSP3_7.0,2050s	39.47	2.26	41.73	0.11	0.27%
	SSP3_7.0,2090s	39.49	2.24	41.73	0.11	0.27%
	SSP5_8.5,2050s	39.49	2.24	41.73	0.11	0.27%
	SSP5_8.5,2090s	39.50	2.23	41.73	0.11	0.27%

In the Southwest Tea Region, the current overlap between primary habitability areas and forest land is 50.41 × 10⁴ km^2^, showing an overall increasing trend under future scenarios. By the 2090s under the SSP5–8.5 scenario, the overlap increases to 51.28 × 10⁴ km^2^, representing a growth of 0.88 × 10⁴ km^2^ and a 1.74% rise in proportion. Due to the region’s high forest coverage and complex topography, the expansion of habitability areas is likely to affect existing forest lands, further intensifying pressure on forest resources. It is therefore recommended to prioritize the use of degraded or marginal forest areas for developing eco-friendly tea plantations and to promote agroforestry systems that integrate tea cultivation with forest conservation, thereby mitigating potential ecological conflicts.

In the Jiangnan Tea Region, the current overlap between habitability areas and forest land is 43.07 × 10⁴ km^2^. Under future climate scenarios, the change is relatively moderate, with a maximum increase of 1.73% (reaching 43.82 × 10⁴ km^2^ under SSP5–8.5 in the 2090s). Most newly expanded habitability areas are adjacent to existing plantations, with overall spatial expansion remaining stable. Despite the manageable additional pressure, land use supervision should still be strengthened in hilly zones and agroforestry transition belts to prevent the gradual encroachment of marginal forest areas.

In the Jiangbei Tea Region, habitat suitability is projected to improve significantly. The overlap with forest land increases from the current 5.05 × 10⁴ km^2^ to 6.78 × 10⁴ km^2^ under SSP5–8.5 in the 2090s—an increase of 1.73 × 10⁴ km^2^ or 30.55%. Expansion is concentrated in hilly and mountainous forest zones, especially under high-emission scenarios, where suitable areas shift rapidly northward, posing a heightened risk of forest conversion. It is recommended to predefine expansion boundaries for habitability areas and establish ecological redlines and land-use policies to prevent large-scale forest exploitation.

In the South China Tea Region, the current overlap area is 41.62 × 10⁴ km^2^, which remains largely unchanged under all future scenarios. The maximum increase is only 0.27%, reaching 41.73 × 10⁴ km^2^. As suitability in this region is already near saturation, there is little room for further expansion, and the additional pressure on forest resources is minimal. Management efforts should focus on disaster prevention and ecological optimization, including soil and water conservation, early warning systems, and improving the climate resilience of existing tea plantations.

In conclusion, under climate change, the impact of tea plant habitability area expansion on forest land varies by region. The Southwest and Jiangbei Tea Regions face greater risks of forest encroachment and require stricter spatial planning and ecological regulation. In contrast, the Jiangnan and South China Tea Regions exhibit more stable habitability patterns, making them more suitable for advancing eco-friendly development strategies that balance industrial growth with forest conservation.

## 5. Discussion

### 5.1. key environmental factors influencing the distribution of primary habitability areas for tea plants

The main factors affecting the potential distribution of suitable habitats for tea plants in China’s four major tea-producing regions include BIO12 (annual precipitation), BIO4 (temperature seasonality), BIO15 (precipitation seasonality), DEM (digital elevation model), TK5–15 (potassium content at 5–15 cm soil depth), TP30–60 (phosphorus content at 30–60 cm), ST (soil thickness), and TN15–30 (nitrogen content at 15–30 cm). Among these, climatic variables are dominant, with the top three factors together contributing over 53% in total. Topographic (DEM) and soil factors together account for half of the top eight, highlighting the multidimensional responses of tea plants to thermal conditions, water availability, and soil nutrients.

Among climatic factors, BIO12 (annual precipitation) shows the highest importance score of 0.245, indicating the strong sensitivity of tea plants to water supply. Studies have shown that adequate precipitation not only influences yield but also significantly enhances the accumulation of bioactive compounds and improves tea quality [55]. BIO4 (temperature seasonality) and BIO15 (precipitation seasonality) also rank highly, reflecting the tea plant’s capacity to adapt to intra-annual variations in temperature and rainfall. Most of eastern China is influenced by a monsoonal climate, with concurrent rainfall in summer and dry winters, which forms a favorable hydrothermal coupling for tea plant growth in southern hilly areas [56].

Among geophysical variables, DEM (elevation) reflects the spatial redistribution of thermal and moisture conditions caused by topographic variation. The southern mountainous regions exhibit large elevation differences and complex slope aspects, creating diverse microclimates. The joint effects of altitude and slope direction significantly shape thermal gradients and moisture redistribution, which are crucial in shaping suitable habitats [57].

Regarding soil variables, TK5–15 (potassium), TP30–60 (phosphorus), TN15–30 (nitrogen), and ST (soil thickness) all demonstrate high importance scores. Potassium and phosphorus are key nutrients that regulate tea plant metabolism and enhance stress tolerance, while adequate soil thickness supports root development and water retention, ensuring the stable growth of tea plants [58].

In summary, the formation of suitable habitats for tea plants results from the synergistic interaction among multiple factors—climate, hydrothermal regimes, topography, and soil conditions. Climate provides the fundamental framework, while topography and soil exert fine-scale regulation at the regional level, offering scientific guidance for future habitat prediction and cultivation zoning.

### 5.2. Impact of future climate warming on the distribution of tea plant habitability areas in china’s four major tea-producing regions

Under future climate warming scenarios, the overall distribution of tea plant habitability areas in China shows a clear trend of northward expansion. In particular, the area of High Habitability Areas increases under all six future climate pathways (SSP1–2.6, SSP3–7.0, and SSP5–8.5 for the 2050s and 2090s). This indicates that tea cultivation zones in China may expand spatially in the future, potentially increasing the total area suitable for cultivation and strengthening the country’s comparative advantage in the global tea industry.

Regionally, the High Habitability Areas in the Jiangnan Tea Region and Jiangbei Tea Region expand significantly, offering new opportunities for boosting tea production capacity and regional economic growth. In contrast, the South China Tea Region shows relatively stable suitability, but greater attention should be paid to extreme climatic events such as heatwaves and heavy rainfall, which may affect both yield and quality. The Southwest Tea Region, with its broad altitudinal range, complex terrain, and diverse hydrothermal resources, demonstrates strong adaptive capacity and is expected to maintain its core position in future tea production.

However, the expansion of suitable areas driven by climate warming does not necessarily imply a reduced risk for the tea industry. From a biological perspective, the economic lifespan of cultivated tea plants typically ranges from 40 to 60 years [59,60], meaning that even plantations currently located in High Habitability Areas may face limitations in future productivity. Therefore, despite the projected expansion of high habitability zones, the potential overestimation of suitability cannot be ignored. Future studies should incorporate factors such as pest and disease risks, human disturbances, and policy interventions to improve the comprehensiveness and robustness of spatial predictions. This will provide stronger support for scientific planning of tea cultivation zones and for developing effective ecosystem protection strategies.

### 5.3. Tea industry expansion and biodiversity conservation strategies under climate change

Global climate warming is significantly reshaping the spatial patterns of suitable habitats for tea plants. While the northward shift and expansion of Primary Habitability Areas create new development opportunities and potential economic benefits for the tea industry, they may also exert continuous pressure on forest ecosystems, thereby threatening regional biodiversity security. Previous studies have indicated that future newly suitable areas often spatially overlap with existing forests and nature reserves, especially in high-altitude and ecologically sensitive zones [61,62].

This study further reveals that, under future climate scenarios, the expansion of tea plant habitability areas in the Jiangbei Tea Region and Southwest Tea Region results in the most significant forest encroachment. In the Jiangbei Tea Region, the total overlap between High Habitability Areas and Habitability Areas and forest land reaches 6.78 × 10⁴ km^2^ under the SSP5–8.5 scenario in the 2090s—an increase of 30.55% compared to the current level. In the Southwest Tea Region, the overlap increases from 50.41 × 10⁴ km^2^ to 51.28 × 10⁴ km^2^, a growth of 0.88 × 10⁴ km^2^. These two regions are predominantly mountainous and hilly, with high forest coverage, making it difficult for habitability area expansion to avoid forest lands—thus posing significant risks of ecological conflict.

Additionally, the Jiangbei Tea Region faces dual pressures of spatiotemporally uneven precipitation and increasing drought risks. The expansion of suitable areas will further intensify demand for agricultural irrigation water [63–66]. While the Jiangnan and South China Tea Regions show relatively stable future expansions, their widespread forest coverage and rich biodiversity mean that tea plantation development may still disturb local ecosystems. For example, previous studies have found that tea plantation expansion may reduce the activity range of large animals such as Asian elephants, leading to habitat fragmentation or even displacement [67,68].

Therefore, in the context of climate change, it is crucial to remain alert to the potential ecological impacts of expanding tea plant habitability areas, and to integrate tea industry planning with ecological conservation strategies from the outset. The following measures are recommended:

- Prioritize the layout of new tea plantations in degraded forestlands or agroforestry transition zones with lower ecological sensitivity;
- Promote the construction of eco-friendly tea plantations and develop “forest–tea integrated systems” to alleviate land use conflicts;
- Strengthen the integration of tea industry planning with spatial control systems such as ecological redlines and nature reserve zoning;
- Conduct baseline ecological assessments and environmental impact evaluations in high-risk areas in advance.

Only by achieving a balance between tea industry development and biodiversity conservation can we address the challenges of climate warming while promoting the sustainable transformation and high-quality development of tea production.

### 5.4. Model discussion and uncertainty analysis

This study employed five mainstream machine learning models to simulate and compare the habitat suitability of tea plants, ultimately selecting the Random Forest model for spatial prediction. Different models exhibit varying responses to marginal samples and extreme environmental conditions, which may affect the clarity of prediction boundaries and local accuracy. For example, the MaxEnt model performs better in areas with sparse data, while XGBoost demonstrates advantages in handling high-dimensional data.

In the model construction process, variable selection and preprocessing have a significant impact on prediction performance. Although Pearson correlation analysis and variable importance screening were applied to reduce the risk of multicollinearity, micro-environmental differences in certain regions may not have been fully captured, affecting the accuracy of spatial extrapolation. In addition, in some regions, limited sample sizes and uneven spatial distribution—particularly in areas with high ecological heterogeneity—may reduce prediction accuracy. To address this, future research could increase field sampling, introduce data augmentation methods, and conduct robustness checks (e.g., different random partitions, alternative algorithm comparisons, or cross-validation) to verify the stability of results under varying data conditions, thereby improving prediction reliability. Although pseudo-absence samples were generated using the “disk” strategy to minimize spatial overlap and enhance independence, their quantity and spatial distribution may still influence model performance.

The predictor variables in this study encompassed three categories of natural environmental factors: climate, topography, and soil. However, socio-ecological drivers such as pest and disease occurrence, human activities, and land-use change were not included. Previous research has indicated that climate change may indirectly affect tea yield and quality by altering pest distributions and habitat suitability [69]. Future studies could incorporate these socio-ecological variables into the modeling framework to provide more comprehensive suitability assessments.

Furthermore, the future scenario projections in this study were based solely on a single climate model (BCC-CSM2-MR), which may have certain limitations in simulating localized extreme events. Future work is recommended to combine multi-model ensembles to enhance robustness and reliability, and to explore integrating socio-economic variables—such as policy regulation and planting intentions—together with natural factors, to better support adaptive management and planning for the tea industry.

## 6. Conclusions

Based on 376 presence records of tea plants and 14 variables covering climate, soil, and topography, this study employed five machine learning models, including Random Forest, to predict the current distribution and future changes in suitable habitats for tea plants across China’s Four Major Tea-Producing Regions. The main conclusions are as follows:

The Random Forest model showed the best overall performance. The dominant environmental variables shaping the distribution of suitable habitats for tea plants include annual precipitation, temperature annual range, precipitation seasonality, soil thickness, as well as phosphorus and potassium content in shallow soil layers, highlighting tea plants’ sensitive response to hydrothermal and nutrient conditions.

Under current climate conditions, the area of High habitability area across the Four Major Tea-Producing Regions is 1474.2 × 10⁴ km^2^, and the area of Habitability area is 1965.9 × 10⁴ km^2^, together accounting for 86.84% of the total tea-producing area. These areas are mainly concentrated in regions with favorable ecological conditions and exhibit relatively stable spatial patterns.

Under future climate scenarios, changes in habitability levels vary across regions. Both the Jiangnan Tea Region and Jiangbei Tea Region show significant improvement in habitability. In particular, the proportion of High habitability area in the Jiangbei Tea Region increases from 6.46% to 23.13%. The Southwest Tea Region remains relatively stable overall, though the Middle habitability area rises to 178.5 × 10⁴ km^2^ under the SSP5–8.5 (2090s) scenario. The South China Tea Region consistently maintains a High habitability area pattern, with its proportion ranging from 94.16% to 95.20%, while the Habitability area fluctuates slightly.

While climate warming promotes the expansion of suitable habitats, it also increases pressure on forest land. Under the SSP5–8.5 (2090s) scenario, the overlap between primary habitability areas (High habitability area + Habitability area) and forest land increases by 8.8 × 10⁴ km^2^ (+1.74%) in the Southwest Tea Region and by 17.3 × 10⁴ km^2^ (+30.55%) in the Jiangbei Tea Region. Changes in the Jiangnan and South China Tea Regions are relatively minor, with increases not exceeding 1.73%.

In summary, future climate change will promote both the structural optimization and northward expansion of tea plant suitability across China’s Four Major Tea-Producing Regions, particularly in the Jiangbei and Jiangnan Tea Regions. At the same time, it is necessary to balance the expansion of suitable habitats with forest conservation by formulating region-specific adaptation and management strategies to ensure ecological security and the sustainable development of the tea industry.
